# The submental flap for oral cavity reconstruction: Extended indications and technical refinements

**DOI:** 10.1186/1758-3284-3-51

**Published:** 2011-12-20

**Authors:** Ayman A Amin, Mostafa A Sakkary, Ashraf A Khalil, Mohammmed A Rifaat, Sherif B Zayed

**Affiliations:** 1 Surgery department, National Cancer Institute (NCI), Kasr El-Aini St.,Fom El-Khalig, Cairo 11796, Egypt; 2 Plastic Surgery Department, Kasr El-Aini School of Medicine, Kasr El- Aini St., Cairo University, Cairo, Egypt

**Keywords:** submental, flap, oral, composite resection, mandibulectomy

## Abstract

**Background and purpose:**

The submental flap is gaining popularity as a simple technique for reconstruction of small to moderate size defects of the oral cavity. However, its role in composite defects involving the jaw is not clearly defined. Indeed, controversy exists about the flap's interference with an oncologically sound neck dissection

**Patients and Methods:**

A total of 21 patients with oral cavity cancers over a three year period were included. All patients underwent surgical resection and immediate reconstruction with submental flap except one patient who had delayed reconstruction with reversed flap. The flap was used for reconstruction of intra-oral soft tissue defect in 13 patients and composite defects in 8 patients.

**Results:**

Of 21 patients 12 were males and 9 were females, age ranged from 32 to 83 years. The primary tumor sites included buccal mucosa (7), tongue (4), alveolar margin (3), floor of mouth (5) and lip (2). Eventually in this study, we adopted completing the neck dissection first before flap harvest. Complete flap loss occurred in 2 whereas 3 patients had partial flap loss. Follow up ranged from 3 to 44 months, one patient died from metastatic disease. Four patients developed neck recurrences.

**Conclusion:**

The submental flap is a valid option for reconstruction of intra-oral soft tissue as well as composite oral defects particularly in elderly patients. However, oncologically sound neck dissection should be assured.

## Background

Oral cavity cancer is the sixth most common cancer worldwide, and comprises 30% of all head and neck cancers. Oral cancer occurs most commonly in middle-aged and elderly individuals [1].

Most tumors of the oral cavity are squamous cell carcinomas (SCC), but other histological types such as minor salivary gland carcinomas, lymphomas and melanomas may rarely occur. The presence of nodal metastases is the most significant predictor of adverse outcome in head and neck SCC [2].

Surgery has been the mainstay for primary management of oral cavity cancer, while radiotherapy is offered postoperatively to patients at high risk for loco regional recurrence. The excision entails removal of the tumor with a margin of at least 1-1.5 cm. Neck dissection is simultaneously done for either clinically evident nodal disease or for large primary tumors or tumors with a depth of invasion greater than 4 mm. The prognosis for early lesions (T1 and T2) of the oral cavity is good, with a 5-year survival of 80% to 90%. Survival for advanced lesions (T3 and T4) can only range from 30%to 60% [3].

Surgical excision of larger lesions usually creates a two dimensional or three dimensional defects. The reconstruction of such defects has a significant impact on the quality of life for oral cancer patients [4]. Split thickness skin grafts, loco- regional flaps, and free flaps have been used to reconstruct oral cavity defects. Skin grafts may be useful for superficial defects, but they have their limitations [5]. Pectoralis major myocutaneous flap and deltopectoral flap have the disadvantages of being too bulky, have a limited reach and may require a second session for refashioning and division of the pedicle. A variety of local flaps such as Nasolabial flap, Sternocleidomastoid flap and the Platysma flap, have been used, but they are either unreliable or of limited versatility in terms of coverage of intraoral defects. Free flaps such as the radial forearm or the anterolateral thigh (ALT) flaps have became the first choice in the last two decades and are still currently used with great success in reconstructing extensive intra-oral defects. However they need trained personnel, microsurgical setup, and are usually associated with an increased operative time and a longer hospital stay (Poster presentation) [6].

The submental artery flap was first described by Martin et al [7] in 1993. The earliest reported use of this flap for reconstruction in oral carcinoma was by Sterne and Hall [8] in 1996. Since it was described, the flap has been extensively used for reconstruction of small to moderate size oral cavity soft tissue defects [9-13]. However, its role in composite oral cavity defects has not been clearly described. In addition, controversy exists about its interference with neck dissection.

In this article we have evaluated the reliability of this flap in reconstruction of small to medium sized soft tissue defects of the oral cavity as well as composite defects.

## Methods

From May 2007 to October 2010 at the National Cancer Institute and Cairo Teaching Hospital, Egypt, a total of 21 patients with oral cavity carcinoma presented to the surgery department for the resection of their tumors and have been offered reconstruction of the resultant defects with the submental artery flap. Elderly patients, patients preferring neck donor site, and those with medical co-morbidities precluding the option of free tissue transfer to be done safely, were included in this study. Patients with nodal stage more than N1 were excluded from the study. Flap viability, complications, functional and cosmetic results as well as loco-regional control rate were all evaluated.

All of our patients were Egyptian Semitic Whites. The age of patients at presentation ranged from 32 to 83 years (mean is 59 years). Out Of the twenty one patients, there were 12 males and 9 females. Six male patients are smoker, and none of the patients was alcoholic. Co morbid diseases were present in four patients and the ASA Physical Status scoring ranged from 1 to 3.

The main presenting symptom in 17 patients was an intraoral ulcer that failed to respond to medical treatment by the referring physician. The remaining four patients presented with local recurrence after previous surgery and radiotherapy for oral cancer. All patients have preoperative histological diagnosis (table 1). The buccal mucosa was the most common primary site involved (33.3%), followed by the floor of mouth (table 2). The lesions were staged clinically as stage T2 (n = 9), T3 (n = 9), and T4 (n = 3). All of our patients were clinically N0, and all patients were non metastatic (M0) at presentation.

**Table 1:** Types of pathology

Pathology	Number of patients	(%)
- Squamous cell carcinoma	17	80.96
- Microinvasive SCC	2	9.52
- Adenoid cystic carcinoma	1	4.76
- peripheral ameloblastoma	1	4.76

**Total**	21	100%

**Table 2:** Primary tumor sites:

Site	Number of patients	(%)
- Buccal mucosa	7	33.4
- Floor of mouth	5	23.8
- Tongue	4	19.0
- Alveolar margin	3	14.3
- Lip	2	9.5

**Total**	21	100%

Consent was obtained from patients after full explanation of the surgical procedure, the likely outcome and the potential complications that may occur. Written informed consent was obtained from the patient for publication of this case report and accompanying images. A copy of the written consent is available for review by the Editor-in-Chief of this journal. The study proposal has been approved by our research and ethical committee.

### Surgical Technique

The patient lies supine with the head extended and turned to the opposite side.

Loup magnification is used

#### Flap design

An ellipse of skin is outlined in the submental area across the midline. The upper incision is made 1.5 cm below the mandible in the midline and 3.5 cm below the angles of the mandible on both sides. The maximal width of the flap is determined by a pinch test in order to close the donor site primarily. The length of the flap is designed according to the size of the defect and may span from one mandibular angle to the other if necessary. The skin paddle may also be designed to accommodate unilateral or bilateral neck dissection.

#### Neck dissection

this starts first, taking extreme caution to preserve the facial vessels. Then following completion of the neck dissection, flap harvesting starts. This approach should assure an oncologically safe procedure. On approaching the submandibular triangle, the facial artery and vein are carefully dissected away from the submandibular gland by ligating the branches going to the gland and preserving the submental vessels. In case bilateral neck dissection is needed, the flap should be harvested on the less involved side of the neck which should be completed first.

#### Harvesting the flap

Flap dissection begins from the contralateral side of the pedicle in the subplatysmal plane. When dissection reaches the midline, care is taken to identify and dissect the submental artery and vein that course along the medial margin of the anterior belly of the digastric muscle. Occasionally a strip of the myelohyoid muscle is included in the flap. It is detached from the mandible and the hyoid, and is bluntly dissected off the ipsilateral geniohyoid muscle. This results in complete mobilization of the flap.

A generous tunnel can then be created between the defect and the donor site. The flap is routed medial to the mandible when the defect involves the floor of the mouth, the base of the tongue, the tonsillar fossa, or the retromolar trigone. Alternatively, the flap is routed lateral to the mandible for defects that involve the buccal mucosa. The portion of the flap traversing the tunnel is deepithelialized and the flap is insetted. The donor site is then closed primarily in layers.

To achieve even greater mobility, the flap can be converted to ***a reverse flow flap ***based on retrograde flow through the facial vessels by dividing these vessels proximal to the origin of the submental vessels. Ryle nasogastric tube was inserted in all cases and used for immediate post-operative feeding, for ten days or until there is no evidence of wound breakdown or fistula.

## Results

All patients underwent surgical resection and immediate reconstruction with the classical submental flap except one patient who had delayed reconstruction with a reversed flap. The largest skin paddle size taken in our series was 12 × 5 cm.

The flap was used for reconstruction of intra-oral soft tissue defect in 13 patients and composite defects in 8 patients. Table (3) shows data of patients with the composite defects.

**Table 3:** cases with composite defects

Patient	Age	1ry site	TNM stage	Extent of composite resection	Type & result of neck dissection	Pathology	Complications	Postoperative radiotherapy
Case 1	65	Ant. Floor of mouth	T2N0M0	Floor of mouth + marginal mandibulectomy	Bilateral SOHND ve	SCC	--	Yes

Case 2	82	Lower alveolar margin	T4N1M0	Segmental mandibulectomy	Ipsilateral MRND +ve 1/8	SCC	--	No

Case 3	47	Buccal mucosa	T3N0M0	Buccal mucosa + upper alveolar margin + partial maxillectomy	Ipsilateral MRND -ve	SCC	---	Yes

Case 4	47	Rec. lower lip After Rth	T4N0M0	Submental flap for total lower lip+ free fibula or mandible and floor of mouth	Ipsilateral MRND -ve	SCC	---	No

Case 5	51	Rec. buccal mucosa After Rth	T2N0M0	Buccal mucosa + Segmental mandibulectomy+ econstruction Plate + submental flap	Ipsilateral MRN -ve	SCC	Partial external plate Exposure covered by nasolabial flap	No

Case 6	62	Tongue and floor of mouth	T2N1M0	Partial glossectomy + loor of mouth +upper marginal mandibulectomy	Ipsilateral MRND -ve	SCC	---	No

Case 7	33	Central segment mandible	ameloblastoma	Marginal mandibulectomy + loor of mouth	---	Peripheral ameloblastoma	--	No

Case 8	84	Alveolar margin	T4N0M0	Alveolar margin + Segmental mandi-bulectomy + econstruction plate	Ipsilateral MRND -ve	SCC	Partial external plateexposure --- debridement granulation	No

Simultaneous neck dissection was performed in 17 patients. This was completed initially before flap harvest. All patients had an intra-operative microscopic tumor free margins by frozen section. The mean operative blood loss was 300cc (range of 50cc to 800cc).The mean operative time, including resection and reconstruction was 3 hours. Post-operative hospital stay ranged from 3 to 12 Days.

**Figure 1:**
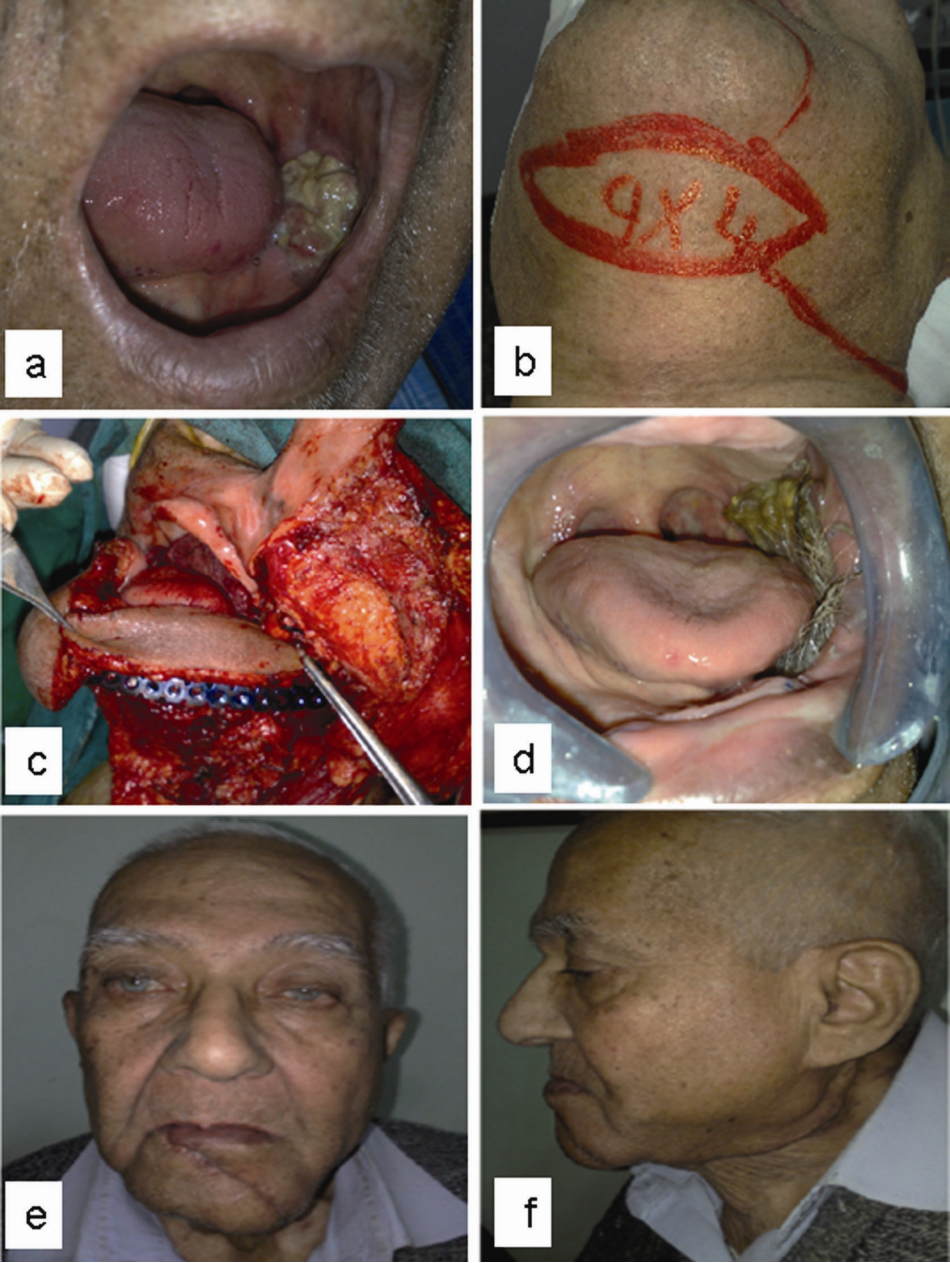
**An 82 years old male with carcinoma of the lower alveolar margin.** a) Preoperative view, b) Preoperative flap design, c) Intraoperative view with the flap covering the reconstruction plate. Three months postoperative views: d) oral view with hair growth. e) anteroposterior view. f) lateral view.

Complete flap loss occurred in 2 patients, one of them died postoperatively from pneumonia after salvage surgery with pectoralis major flap, while the other patient's raw area was left to heal by secondary intention, resulting in mild trismus which has improved with physiotherapy. Partial flap loss occurred in 3 patients, and wounds healed spontaneously. Another patient developed an oro- cutaneous fistula which closed spontaneously with conservative measures. Donor sites healed uneventfully in all cases, leaving inconspicuous scars. Hair growth in the flap persisted in male patients for a variable time and was managed by epilation. Mucosalization of the surface of the flap was noticed after 1 year (figure 1).

Follow up ranged from 7 to 44 months. One patient died from metastatic disease after palliative chemotherapy and another four patients developed ipsilateral nodal neck recurrence. All of those recurrences were in the submandibular triangle at the site of the flap tunnel. Three 3 out of those nodal recurrences had an initial simultaneous neck dissection in whom the flap was harvested first. Nodal recurrence was managed by salvage neck dissection. After adopting the refined technique, we had 0% neck recurrence (table 4).

**Table 4:** Oncologic outcome

Outcome	Number of patients	%
- Alive and free of disease	15	75.0
- Nodal relapse	4	20.0
- Both local and distant relapse	1	5.0
**Total**	20	100%

*the 21th patient died in the immediate postoperative period.

The long term cosmesis and function (speech and swallowing) were good in all the patients. This has been assessed subjectively by the degree of patient satisfaction. All patients were satisfied with the functional outcome except two patients. One of them was the patient who sustained total flap loss and preferred to be treated conservatively, but developed trismus. This showed some improvement with physiotherapy. The other patient suffered restricted tongue mobility and tethering following flap reconstruction of a tongue defect. Surgical release of contracture was done later with some improvement.

## Discussion

Over the past decade, the submental island flap has proved to be a reliable reconstructive option in head and neck surgery, being a simple and rapid flap to harvest [4]. It provides a relatively thin, well vascularized piece of tissue in a single stage operation, and obviates the need for a second stage to divide the pedicle, or sophisticated microsurgical techniques. It has been used after infection, trauma, or tumor extirpation for reconstruction of the mustache and beard area [9], the nose,[10] the pharynx,[11],[12] the palate,[13] and the middle and lower face [13-15]. However up to our knowledge its use in composite intra-oral defects has not been reported before in the western literature.

Including the anterior belly of the digastric muscle in the submental artery flap has been controversial [16,9]. Faltaous and Yetman [16] and Magden et al.[17] found that the main submental artery courses beneath the anterior belly of the digastric muscle in most specimens. However, there is also a superficial branch that runs above the digastric muscle. Indeed, flap survival has not been affected by omitting the muscle [10,15]. In this series, we have included the anterior belly of the digastric muscle. Certainly, including this muscle may have improved flap viability in the cases of the present study, and in the absence of oncologic contraindications, this modification should be considered for future cases. Also, part of the myelohyoid was occasionally incorporated with the flap to protect the perforating vessels and enhance venous drainage, provided that this does not affect the pedicle length.

Though a small flap, yet it successfully covered the reconstruction hard plate securely in cases of segmental mandibulectomy with no single internal extrusion. The flap was used successfully for reconstruction after composite intra-oral resection of upper or lower jaw in 8 patients. Up to our knowledge; this had never been mentioned in literature before.

Chow et al.[18] reported partial loss of two out of 10 flaps. Merten et al. [19] reported loss of one flap in 11 non-irradiated patients. The latter authors mentioned they avoided this flap if the neck had been previously irradiated. In our series, two total and three partial flap losses were recorded. Most reports did not assess the influence of irradiation on flap viability. However, in the experience of Taghinia, and his colleagues [20], preoperative radiotherapy was the most consistent finding in those who suffered flap loss. In the current study, no flap loss occurred in the two patients who had received preoperative radiotherapy. Interestingly, those patients in this study who had postoperative radiation therapy also experienced complication of scar contractures requiring multiple procedures. Thus, in our experience, irradiation significantly predisposes the patient to complications of ischemia and scar contractures.

The probability of facial palsy caused by damage to the facial nerve during surgery for this flap has been reported in literature in the range of 0 to 17%[6]. Temporary marginal mandibular nerve palsy did not develop in this series. Pistre et al.[21] reported one case of temporary marginal mandibular nerve palsy in 31 cases in which the submental flap was used for a variety of defects. Although the latter authors exposed the nerve early in their series, they found that avoidance may be a better approach. Other reports echo similar results [22,23] and highlight the possibility of nerve injury if dissection is not performed carefully. Moreover, the use of nerve stimulators together with careful dissection decrease nerve injury significantly and help preserve the innervations of the supplied muscles [15,20,23].

As regards to the flap's donor site, our results in terms of donor site wound healing and the quality of scarring compare favorably with other reports.

There has been concern in the literature that harvesting this flap can potentially compromise the oncologic treatment of the involved lymph nodes or may result in spreading of the tumor to the recipient area. However, the plane of flap dissection is at the subplatysmal plane, which is also the plane of skin flap elevation by the oncologic surgeon. Thus, if proper anatomical planes are respected, chances of tumor spread can be minimized. A recent report by Chow et al. [18] addressed these oncologic concerns by reviewing 10 cases of submental artery flap reconstruction after resection of aggressive oropharyngeal cancers. Three cancer recurrences were noted that were more likely related to the aggressive nature of the tumors than to the oncologic violation by the flap. In our series, there were 4 nodal recurrences in the early cases. However no single recurrence has developed after we have started completing the neck dissection before flap harvesting. At the latest follow-up, none of the patients in this series showed tumor recurrence in the transferred flap. Other reports correlate well with our findings and lend support to the oncologic safety of this flap [21,23]. Moreover, we have adopted the policy of completion of adequate lymph node dissection before harvesting the flap. The surgeon should be prepared to any oncologic surprise, such as finding suspicious lymph nodes in level I, which could be either seen intraoperatively or proved by frozen section. He or she should never hesitate to extend the lymph node dissection to the opposite side if they the lymph nodes encroach on the midline. The surgeon might even abandon the submental flap and shift to another reconstructive option if this showed to be oncologically necessary. Despite its established safety, we believe that indiscriminate use of this flap for all cancer patients should be discouraged if it is going to jeopardize the cancer operation. This flap should be avoided in those patients with clinically advanced nodal disease in the neck (> N1).

## Conclusion

The submental artery flap is a valid option for reconstruction of small to moderate-sized soft tissue as well as composite oral cavity defects. It represents a reasonable alternative to free flaps particularly in elderly patients. However it has a steep learning curve and oncologic safety must always be a priority.

## List of abbreviations

**ALT**: anterolateral thigh

**ASA**: American Society of Anesthesiologists

**SCC**: squamous cell carcinoma

## Competing interests

The authors declare that they have no competing interests.

## Authors' contributions

AA conceived the study. AA, MS, MR, SZ and AK participated in the design and coordination of the study and performed surgery. MS made substantial contributions to data acquisition and drafted the manuscript. AA, MR and AK were involved in revising the manuscript. All authors read and approved the final manuscript.

## Authors' information

**1- Ayman Abd Elwahab Amin**., MD, Professor of surgical oncology and microsurgery, Surgery Department, National Cancer Institute, Cairo University, Cairo, Egypt.

**2- Mostafa Abd Eltawab Sakkary**, MD, Lecturer of surgical oncology, Surgery Department, National Cancer Institute, Cairo University, Cairo,

3- **Mohammed Ahmed Rifaat: **MD, FRCS, EBOPRAS Assistant Professor of surgical oncology and Reconstruction, Surgery Department, National Cancer Institute, Cairo University, Cairo,

4- **Sherif Bahaa Zayed**., MD, Lecturer of surgical oncology, Surgery Department, National Cancer Institute, Cairo University, Cairo, Egypt

**5- Ashraf Abolfotooh Khalil**., MD, Lecturer of Plastic Surgery, Plastic Surgery Department, Kasr El-Aini School of Medicine, Cairo University, Cairo, Egypt
